# Decline of Humoral Responses against SARS-CoV-2 Spike in Convalescent Individuals

**DOI:** 10.1128/mBio.02590-20

**Published:** 2020-10-16

**Authors:** Guillaume Beaudoin-Bussières, Annemarie Laumaea, Sai Priya Anand, Jérémie Prévost, Romain Gasser, Guillaume Goyette, Halima Medjahed, Josée Perreault, Tony Tremblay, Antoine Lewin, Laurie Gokool, Chantal Morrisseau, Philippe Bégin, Cécile Tremblay, Valérie Martel-Laferrière, Daniel E. Kaufmann, Jonathan Richard, Renée Bazin, Andrés Finzi

**Affiliations:** a Centre de Recherche du CHUM, Quebec, Canada; b Département de Microbiologie, Infectiologie et Immunologie, Université de Montréal, Montreal, Quebec, Canada; c Department of Microbiology and Immunology, McGill University, Montreal, Quebec, Canada; d Affaires Médicales et Innovation, Héma-Québec, Montreal, Quebec, Canada; e CHU Ste-Justine, Montreal, Quebec, Canada; f Département de Médecine, Université de Montréal, Montreal, Quebec, Canada; Columbia University Medical Center; Columbia University/ HHMI

**Keywords:** coronavirus, COVID-19, SARS-CoV-2, Spike glycoproteins, RBD, ELISA, IgA, IgM, IgG, neutralization, cross-reactivity, convalescent plasma

## Abstract

In the absence of effective vaccines and with limited therapeutic options, convalescent plasma is being collected across the globe for potential transfusion to coronavirus disease 2019 (COVID-19) patients. The therapy has been deemed safe, and several clinical trials assessing its efficacy are ongoing. While it remains to be formally proven, the presence of neutralizing antibodies is thought to play a positive role in the efficacy of this treatment. Indeed, neutralizing titers of ≥1:160 have been recommended in some convalescent plasma trials for inclusion. Here, we performed repeated analyses at 1-month intervals on 31 convalescent individuals to evaluate how the humoral responses against the severe acute respiratory syndrome coronavirus 2 (SARS-CoV-2) Spike glycoprotein, including neutralization, evolve over time. We observed that the levels of receptor-binding-domain (RBD)-specific IgG and IgA slightly decreased between 6 and 10 weeks after the onset of symptoms but that RBD-specific IgM levels decreased much more abruptly. Similarly, we observed a significant decrease in the capacity of convalescent plasma to neutralize pseudoparticles bearing wild-type SARS-CoV-2 S or its D614G variant. If neutralization activity proves to be an important factor in the clinical efficacy of convalescent plasma transfer, our results suggest that plasma from convalescent donors should be recovered rapidly after resolution of symptoms.

## Abstract (precis)

While waiting for an efficient vaccine to protect against SARS-CoV-2 infection, alternative approaches to treat or prevent acute COVID-19 are urgently needed. Transfusion of convalescent plasma to treat COVID-19 patients is currently being explored; neutralizing activity in convalescent plasma is thought to play a central role in the efficacy of this treatment. Here, we observed that plasma neutralization activity decreased a few weeks after the onset of the symptoms. If neutralizing activity is required for the efficacy of convalescent plasma transfer, our results suggest that convalescent plasma should be recovered rapidly after the donor recovers from active infection.

## OBSERVATION

Until an efficient vaccine to protect against severe acute respiratory syndrome coronavirus 2 (SARS-CoV-2) infection becomes available, alternative approaches to treat or prevent acute coronavirus disease 2019 (COVID-19) are urgently needed. A promising approach is the use of convalescent plasma containing anti-SARS-CoV-2 antibodies (Abs) collected from donors who have recovered from COVID-19 (1). Convalescent plasma therapy has been successfully used in the treatment of SARS, Middle East respiratory syndrome (MERS), and influenza virus H1N1 pandemics and was previously shown to be associated with improvement of clinical outcomes (2–4). Experience to date has shown that the passive transfer of convalescent plasma to acute COVID-19 patients is well tolerated and presented some hopeful signs (5–9). In one study, the convalescent plasma used had high titers of IgG to SARS-CoV-2 (at least ≥1:640), which correlated positively with neutralizing activity (10). While it remains to be formally demonstrated, neutralizing activity is considered an important determinant of convalescent plasma efficacy (11) and regulatory agencies have been recommending specific thresholds for qualifying convalescent plasma prior to its release. While neutralizing function has been associated with protection against reinfection in rhesus macaques (12), other antibody functions may be relevant for controlling an acute infection and should be examined to better understand the correlates of convalescent plasma-mediated efficacy (7).

It was recently reported that the humoral responses against SARS-CoV-2 are built rapidly, peaking at week 2 or week 3 after the onset of symptoms but steadily decreasing thereafter (13–15). Moreover, in a previous cross-sectional study, we reported that the neutralization capacity decreased between the third and the sixth week after the onset of symptoms (14). Since convalescent patients are generally required to wait for 14 days after recovery to start plasma donations and since they may donate plasma multiple times in the ensuing weeks, most donations are likely to occur even later than this. Whether the neutralization capacity of convalescent plasma is stabilized after 6 weeks or decreases further remains unknown. To address this issue, which might have practical implications for the selection of plasma from convalescent donors, we analyzed serological samples from 31 convalescent donors that were collected at 6 and 10 weeks after the onset of symptoms.

All of the convalescent donors initially tested positive for SARS-CoV-2 by reverse transcriptase PCR (RT-PCR) on nasopharyngeal specimens, with complete resolution of symptoms for at least 14 days before blood sampling. The average age of the donors (22 males and 9 females) was 46 years. We collected plasma samples from each individual at two time points: 6 weeks after the onset of symptoms (baseline; median, 43 days) and 4 weeks after (1 month; median, 74 days after the onset of symptoms) (Table 1).

**TABLE 1:** Cohort characteristics

Median no. of days (range) after onset of symptoms and first sample collection: baseline	Median no. of days (range) after onset of symptoms and second sample collection (1 mo)	Avg age of individuals in yrs (range)	No. of individuals	
			Male (*n*)	Female (*n*)
43 (16–60)	74 (44–87)	46 (20–67)	22	9

**FIG 1:**
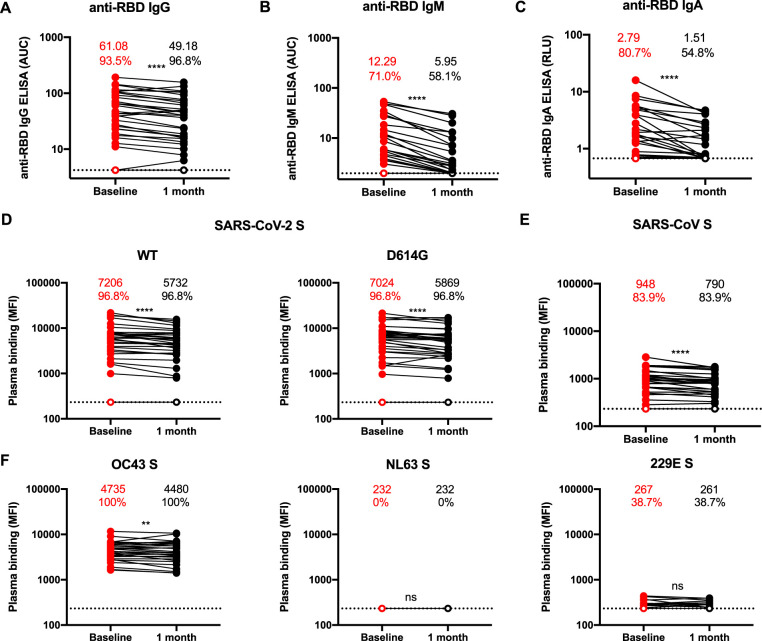
SARS-CoV-2 S-specific and RBD-specific antibody levels decrease over time. (A to C) Indirect ELISA was performed using recombinant SARS-CoV-2 RBD and incubation with plasma samples recovered at baseline (6 weeks after the onset of symptoms; red circle) and 1 month later (black circle). Anti-RBD antibody binding was detected using (A) anti-IgG-HRP (anti-IgG horseradish peroxidase), (B) anti-IgM-HRP, or (C) anti-IgA-HRP. Relative light unit (RLU) values obtained with bovine serum albumin (BSA) (negative control) were subtracted and further normalized to the signal obtained with the anti-RBD CR3022 monoclonal antibodies (MAb) present in each plate. The graphs shown in panels A to C represent (A and B) the areas under the curve (AUC) calculated from RLU obtained with serial plasma dilutions or (C) the normalized RLU for one plasma dilution (1:500). (D to F) Cell surface staining of 293T cells expressing full-length Spike (S) from different HCoVs, including (D) SARS-CoV-2 or its D614G counterpart; (E) SARS-CoV; and (F) OC43, NL63, and 229E with plasma samples recovered at baseline (6 weeks after the onset of symptoms) and 1 month later. The graphs shown in panels D to F represent median fluorescence intensities (MFI). In panels A to F, undetectable levels are represented as white symbols, and limits of detection are plotted. The average numbers and percentages of positive samples are indicated at the top of each panel. Statistical significance was tested using Wilcoxon matched-pair signed-rank tests (ns, not significant; **, *P* < 0.01; ****, *P* < 0.0001).

We first evaluated the presence of receptor-binding-domain (RBD)-specific IgG, IgM, and IgA antibodies by enzyme-linked immunosorbent assay (ELISA) as we had recently described (14). In agreement with a recent report (16, 23), we observed that all RBD-specific IgG, IgM, and IgA titers significantly decreased between 6 and 10 weeks after the onset of symptoms. We noted that IgM and IgA titers diminished significantly more abruptly than IgG titers (Fig. 1). Accordingly, the proportions of convalescent individuals presenting detectable titers of IgM and IgA decreased by ∼13% and ∼25%, respectively, at 10 weeks after the onset of symptoms (Fig. 1B and C) whereas the percentage of infected individuals presenting detectable titers of IgG remained stable (Fig. 1A).

We next used flow cytometry to examine the ability of convalescent plasma to recognize the full-length SARS-CoV-2 Spike protein expressed at the cell surface. Briefly, 293T cells expressing SARS-CoV-2 S glycoproteins were stained with plasma samples, followed by incubation with secondary antibodies recognizing all antibody isotypes. Since the SARS-CoV-2 strain circulating in Europe and North America has the D614G mutation (17), we also evaluated recognition of this variant by flow cytometry. As presented in Fig. 1D, convalescent plasma from 96.8% of donors (all but one) recognized both SARS-CoV-2 S variants (wild type [WT] and D614G) at baseline. While this percentage was found to have remained stable 4 weeks later, the level of recognition (mean fluorescence intensity [MFI]) was significantly diminished for both WT and D614G S-expressing cells, indicating that Spike-reactive antibodies were less abundant in convalescent plasma collected at this later time point. Interestingly, the MFI values were almost identical for the cells expressing the WT S and those expressing the D614G variant S (7,206 and 7,024, respectively; Fig. 1D), suggesting that the mutation did not significantly affect the S conformation. In agreement with recent work, we observed that SARS-CoV-2-elicited antibodies cross-reacted with human sarbecoviruses (14) (SARS-CoV; Fig. 1E) and with another betacoronavirus (OC43) whereas no cross-reactive antibodies to alphacoronavirus (NL63 and 229E) S glycoproteins (Fig. 1F) were detected. Levels of cross-reactive antibodies recognizing SARS-CoV and OC43 S glycoproteins decreased between the two time points, following a trend similar to that shown by the SARS-CoV-2 S-reactive antibodies (Fig. S1).

**FIG 2:**
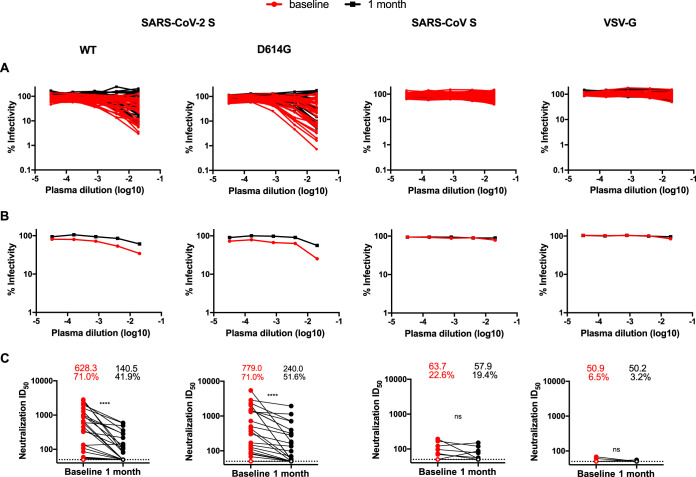
Neutralizing activity of convalescent plasma decreases over time. (A) Pseudoviral particles coding for the luciferase reporter gene and bearing SARS-CoV-2 S glycoprotein or its D614G counterpart, SARS-CoV S glycoprotein, or VSV-G glycoprotein were used to infect 293T-ACE2 cells. Pseudoviruses were incubated (37°C, 1 h) with serial dilutions of plasma samples recovered at baseline (6 weeks after the onset of symptoms) or collected 1 month later prior to infection of 293T-ACE2 cells. Infectivity at each dilution was assessed in duplicate, and data are shown as the percentage of infection without plasma for each pseudovirus. (B) The median of neutralization for baseline (red) or 1-month (black) plasma samples is shown. (C) Neutralization half-maximal inhibitory plasma dilution (ID_50_) values were determined using a normalized nonlinear regression with GraphPad Prism software. Undetectable levels (ID_50_ < 50) are represented as white symbols. The mean neutralizing titers and the proportions (%) of neutralizers (patients with an ID_50_ value over 50) are shown above the graphs. Statistical significance was tested using Wilcoxon matched-pair signed-rank tests (ns, not significant; ****, *P* < 0.0001).

We next measured the capacity of plasma samples to neutralize pseudoparticles bearing WT SARS-CoV-2 S, its D614G variant, or vesicular stomatitis virus G (VSV-G) glycoproteins using 293T cells stably expressing ACE2 as target cells (Fig. 2). Previous studies demonstrated that the neutralizing activity of convalescent plasma measured with this method correlates quantitatively with neutralizing activity measured using an authentic SARS-CoV-2 neutralization assay (18, 19). Neutralizing activity against SARS-CoV-2 WT or D614G S glycoprotein, as measured by the neutralization half-maximum inhibitory dilution (ID_50_), was detected in 71% of patients 6 weeks after the onset of symptoms. While we acknowledge that the sensitivity of any given neutralization assay could affect calculations of the percentage of donors with neutralization activity, we note that the percentage of convalescent plasma with undetectable neutralization titers reported here is similar to what was reported in recent studies (11, 20, 21). SARS-CoV-2 neutralization was specific since no neutralization was observed against pseudoparticles expressing VSV-G (Fig. 2). Neutralizing activity against pseudoparticles bearing the SARS-CoV S glycoprotein was detected in only 25% of convalescent plasma and exhibited low potency, as previously reported (Fig. 2) (14). As recently shown, plasma samples from prepandemic SARS-CoV-2-negative and SARS-CoV-negative individuals showed no neutralization activity against pseudoparticles bearing the SARS-CoV-2 or SARS-CoV Spike protein (not shown). Of note, while we observed enhanced infectivity for the D614G variant compared to its WT SARS-CoV-2 S counterpart (see Fig. S2A in the supplemental material), no major differences in neutralization with convalescent plasma were detected at either time point (Fig. S2B), thus suggesting that the D614G change does not affect the overall conformation of the Spike, in agreement with recent findings (17, 22).

10.1128/mBio.02590-20.2 FIG S1 Decrease in levels of cross-reactive antibodies. Fold decrease (1 month versus baseline) of the capacity of plasma to recognize SARS-CoV-2 S WT, SARS-CoV-2 S D614G, SARS-CoV S, OC43 S, NL63 S, and 229E S glycoproteins expressed at the surface of 293T cells demonstrated by flow cytometry. Statistical significance was tested using Wilcoxon matched-pair signed-rank tests (ns, not significant; **, *P* < 0.01; ****, *P* < 0.0001). Download FIG S1, PDF file, 0.4 MB. Copyright © 2020 Beaudoin-Bussières et al. 2020 Beaudoin-Bussières et al. This content is distributed under the terms of the Creative Commons Attribution 4.0 International license.

10.1128/mBio.02590-20.3 FIG S2 The D614G mutation enhances SARS-CoV-2 infectivity but does not affect its susceptibility to plasma neutralization. (A) Normalized reverse transcriptase levels of pseudoviral particles bearing the SARS-CoV-2 S WT or D614G variant were used to infect 293T/ACE2 cells, and infectivity was measured 48 h later by luciferase activity. The graph shown presents percentages of infectivity relative to pseudoviral particles bearing the SARS-CoV-2 S WT. Statistical significance was tested using Mann-Whitney U tests (****, *P* < 0.0001). (B) Comparison of neutralization ID_50_ levels from pseudoparticles bearing SARS-CoV-2 S WT and SARS-CoV-2 S D614G. Statistical significance was tested using Wilcoxon matched-pair signed-rank tests. ns, not significant. Download FIG S2, PDF file, 0.4 MB. Copyright © 2020 Beaudoin-Bussières et al. 2020 Beaudoin-Bussières et al. This content is distributed under the terms of the Creative Commons Attribution 4.0 International license.

The capacity to neutralize SARS-CoV-2 S WT- or D614G-pseudotyped particles significantly correlated with the presence of RBD-specific IgG, IgM, IgA, and anti-S antibodies (Fig. S3). Interestingly, we observed a pronounced (20% to 30%) decrease in the proportion of convalescent individuals able to neutralize pseudoparticles bearing SARS-CoV-2 S glycoprotein between 6 and 10 weeks after the onset of symptoms. Moreover, with plasma that still neutralized, the neutralization activity significantly decreased between these two time points (Fig. 2C). Interestingly, RBD-specific IgM and neutralizing activity declined more significantly in convalescent plasma over time than RBD-specific IgG, IgA, and anti-S Ab activity (Fig. S4A and B). Moreover, while the loss of neutralizing activity on the WT and D614G pseudoparticles over time correlated with the loss of anti-RBD IgM, IgA, and IgG antibodies, the correlation was higher for IgM than for IgG and IgA (Fig. S4C and D), suggesting that at least part of the neutralizing activity could be mediated by IgM, as recently proposed (13, 14). Therefore, if plasma neutralization activity is shown to be required for protection from SARS-CoV-2 infection, then our results suggest that this protection could be limited in time and that, in the context of vaccination, multiple boosts might be necessary to mount a durable and effective anti-SARS-CoV-2 humoral response.

10.1128/mBio.02590-20.4 FIG S3 SARS-CoV-2 RBD-specific and full-length S-specific antibodies correlate with pseudovirus neutralization. Anti-RBD IgG and IgM levels evaluated by ELISA (A and D), anti-S antibody levels evaluated by flow cytometry (B and E), or anti-RBD IgA levels evaluated by ELISA (C and F) were plotted against the levels of neutralization (ID_50_) of pseudoparticles bearing the SARS-CoV-2 S WT (A, B, and C) or its D614G counterpart (D, E, and F). Statistical analysis was performed using Spearman rank correlation tests. Download FIG S3, PDF file, 0.4 MB. Copyright © 2020 Beaudoin-Bussières et al. 2020 Beaudoin-Bussières et al. This content is distributed under the terms of the Creative Commons Attribution 4.0 International license.

10.1128/mBio.02590-20.5 FIG S4 Decreases in levels of anti-RBD IgM antibodies over time correlate with reduced neutralizing activity. (A and B) Fold decreases in pairs of plasma samples from the 31 individuals over the course of 1 month (1 month over baseline) in levels of anti-SARS-CoV-2 S WT or D614G antibodies quantified by flow cytometry and of anti-RBD antibodies (IgA, IgM, and IgG) quantified by ELISA and fold decrease in neutralization ID_50_ values with pseudoparticles bearing (A) SARS-CoV-2 S WT or (B) SARS-CoV-2 S D614G. (C and D) Correlation between the fold decrease over the course of 1 month in levels of anti-SARS-CoV-2 S WT or D614G antibodies quantified by flow cytometry and anti-RBD (IgA, IgM, and IgG) antibodies quantified by ELISA and fold decrease in neutralization ID_50_ values of pseudoparticles bearing (C) SARS-CoV-2 S WT or (D) SARS-CoV-2 S D614G. For panels A and B, statistical significance was tested using Wilcoxon matched-pair signed-rank tests (**, *P* < 0.01; ***, *P* < 0.001; ****, *P* < 0.0001). (C and D) Statistical significance was tested using Spearman rank correlation tests. Download FIG S4, PDF file, 1.0 MB. Copyright © 2020 Beaudoin-Bussières et al. 2020 Beaudoin-Bussières et al. This content is distributed under the terms of the Creative Commons Attribution 4.0 International license.

In summary, our results indicate that plasma neutralization activity continues decreasing past the sixth week of symptom onset (14). It is currently unknown whether neutralizing activity truly drives the efficacy of convalescent plasma in acute COVID-19. If this were to be found to be the case, our results suggest that efforts should be made to ensure that convalescent plasma is collected as soon as possible after recovery of the donor from active infection.

10.1128/mBio.02590-20.1 TEXT S1 Supplemental materials and methods. Download Text S1, DOCX file, 0.1 MB. Copyright © 2020 Beaudoin-Bussières et al. 2020 Beaudoin-Bussières et al. This content is distributed under the terms of the Creative Commons Attribution 4.0 International license.
