# Supplementary material for: Decline of Humoral Responses against SARS-CoV-2 Spike in Convalescent Individuals
Source: mBio. 2020 Oct 16;11(5):e02590-20. doi: 10.1128/mBio.02590-20 (PMC7569150; doi:10.1128/mBio.02590-20)
Supplement: FIG S1 [file mBio.02590-20-sf001.pdf]

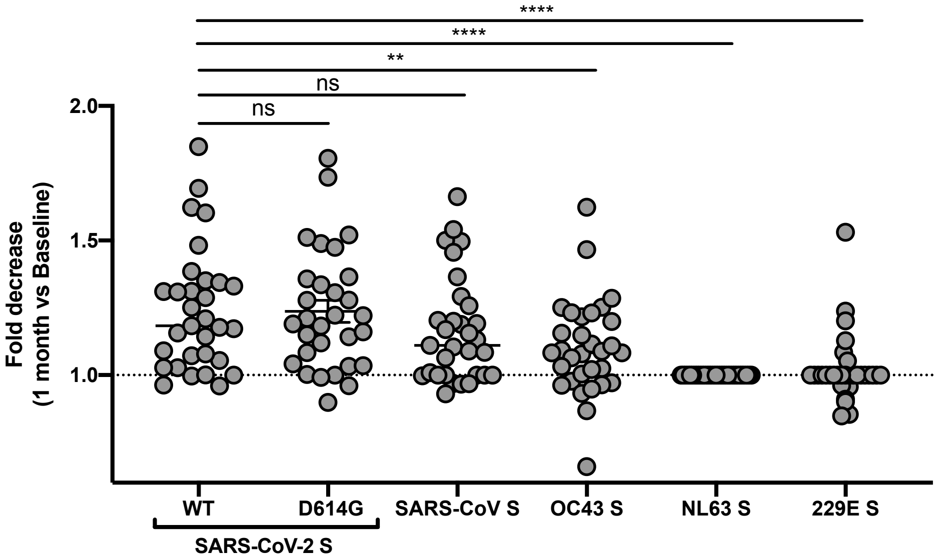

Supplemental Figure 1. Decrease in cross-reactive antibodies
Fold decrease (1 month vs baseline) of the capacity of plasma to recognize by flow cytometry SARS-CoV-2 S WT, SARS-CoV-2 S D614G, SARS-CoV S, OC43 S, NL63 S and 229E S glycoproteins expressed at the surface of 293T cells. Statistical significance was tested using Wilcoxon matched-pairs signed rank tests (ns, not significant; ** $p < 0.01$; **** $p < 0.0001$).
