# Supplementary material for: Decline of Humoral Responses against SARS-CoV-2 Spike in Convalescent Individuals
Source: mBio. 2020 Oct 16;11(5):e02590-20. doi: 10.1128/mBio.02590-20 (PMC7569150; doi:10.1128/mBio.02590-20)
Supplement: FIG S2 [file mBio.02590-20-sf002.pdf]

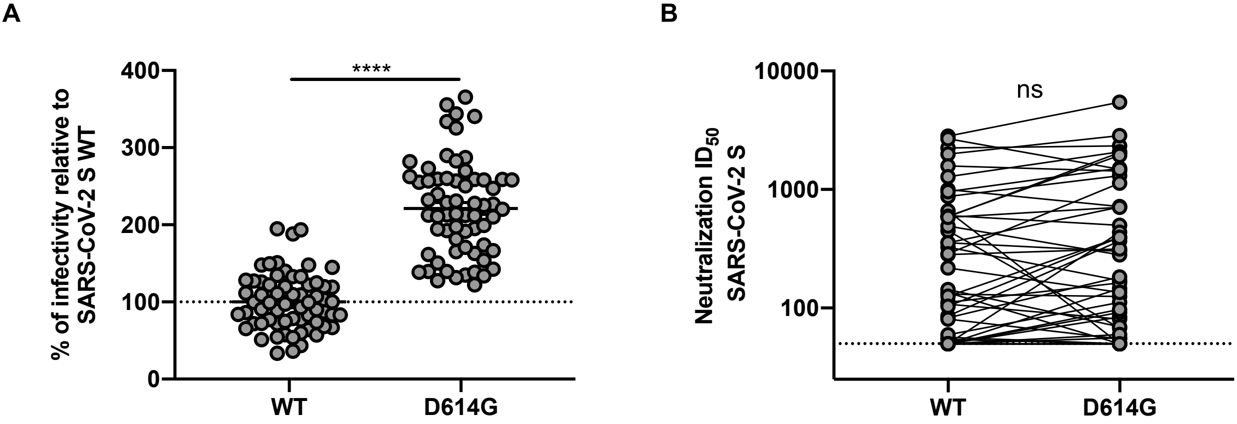

Supplemental Figure 2. The D614G mutation enhances SARS-CoV-2 infectivity but does not affect its susceptibility to plasma neutralization.
(A) Reverse Transcriptase normalized levels of pseudoviral particles bearing the SARS-CoV-2 S WT or D614G variant were used to infect 293T/ACE2 cells and infectivity measured 48h later by luciferase activity. Graph shown represents the percentage of infectivity relative to pseudoviral particle bearing the SARS-CoV-2 S WT. Statistical significance was tested using Mann-Whitney U tests (**** $p < 0.0001$). (B) Comparison between the neutralization ID50 from pseudoparticles bearing SARS-CoV-2 S WT and SARS-CoV-2 S D614G. Statistical significance was tested using Wilcoxon matched-pairs signed rank test. (ns, not significant).
