# Supplementary material for: Decline of Humoral Responses against SARS-CoV-2 Spike in Convalescent Individuals
Source: mBio. 2020 Oct 16;11(5):e02590-20. doi: 10.1128/mBio.02590-20 (PMC7569150; doi:10.1128/mBio.02590-20)
Supplement: FIG S3 [file mBio.02590-20-sf003.pdf]

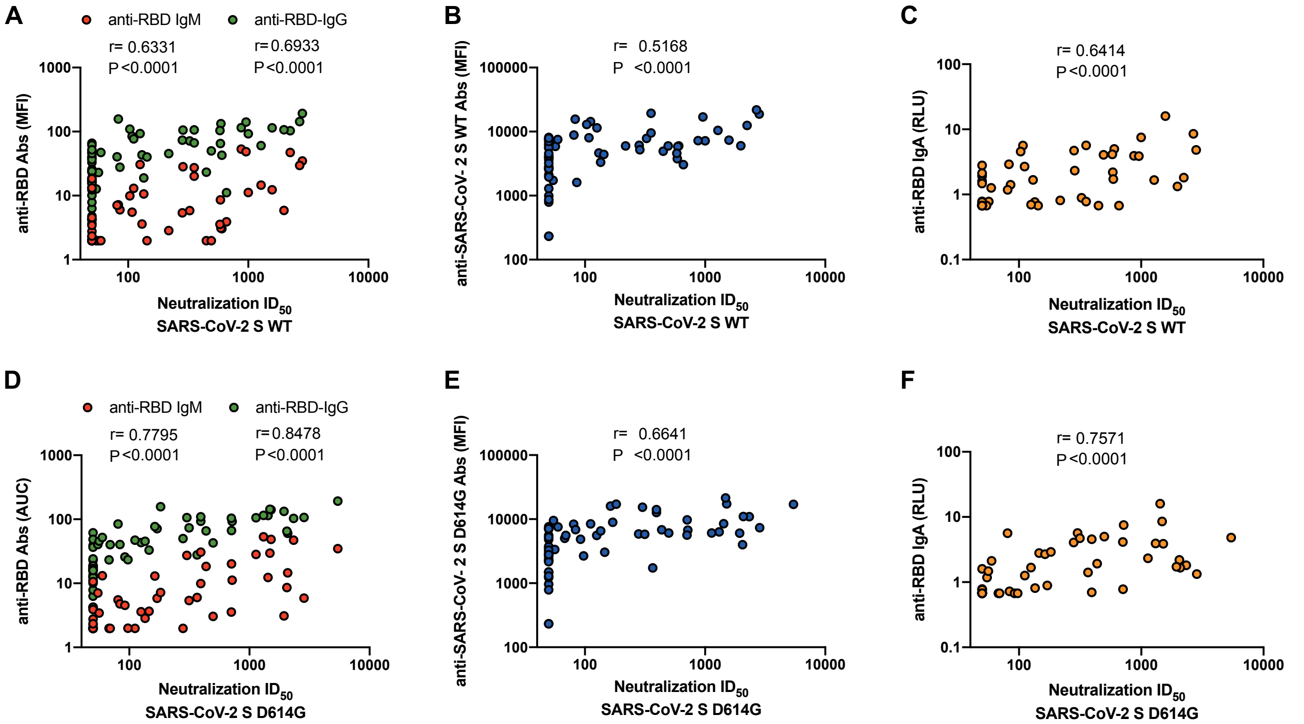

Supplemental Figure 3. SARS-CoV-2 RBD and full length S specific antibodies correlate with pseudoviruses neutralization. Anti-RBD IgG and IgM evaluated by ELISA (A, D) anti-S antibodies evaluated by flow cytometry (B, E) or anti-RBD IgA evaluated by ELISA (C, F) were plotted against the levels of neutralization (ID50) of pseudoparticles bearing the SARS-CoV-2 S (A,B,C) or its D614G counterpart (D,E,F). Statistical analysis was performed using Spearman rank correlation tests.
