# Supplementary material for: Decline of Humoral Responses against SARS-CoV-2 Spike in Convalescent Individuals
Source: mBio. 2020 Oct 16;11(5):e02590-20. doi: 10.1128/mBio.02590-20 (PMC7569150; doi:10.1128/mBio.02590-20)
Supplement: FIG S4 [file mBio.02590-20-sf004.pdf]

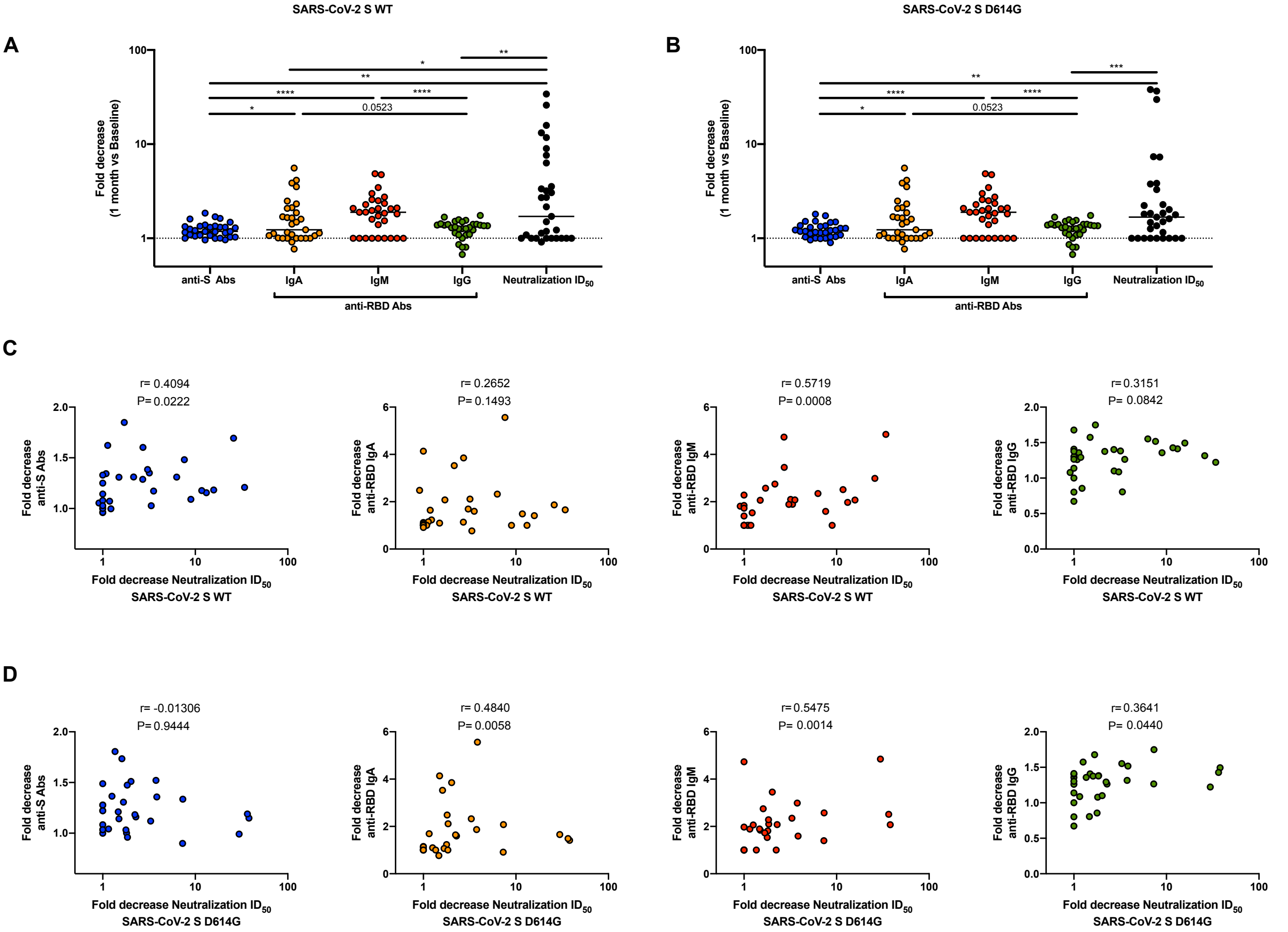

Supplemental Figure 4. Decrease in anti-RBD IgM antibodies over time correlates with reduced neutralizing activity.
Fold decrease of the 31 pairs of plasma over the course of 1 month (1 month over Baseline) of the levels of anti-SARS-CoV-2 S WT or D614G antibodies quantified by flow cytometry, anti-RBD antibodies (IgA, IgM, IgG) quantified by ELISA and of neutralization ID50 with pseudoparticules bearing (A) SARS-CoV-2 S WT or (B) SARS-CoV-2 S D614G. Correlation between the fold decrease over the course of 1 month of anti-SARS-CoV-2 S WT or D614G antibodies quantified by flow cytometry, anti-RBD (IgA, IgM, IgG) antibodies quantified by ELISA and the fold decrease of the neutralization ID50 of pseudoparticules bearing (C) SARS-CoV-2 S WT or (D) SARS-CoV-2 S D614G. (A, B) Statistical significance was tested using Wilcoxon matched-pairs signed rank tests (** $p < 0.01$; *** $p < 0.001$; **** $p < 0.0001$). (C, D) Statistical significance was tested using Spearman rank correlation tests.
