# Supplementary material for: Decline of Humoral Responses against SARS-CoV-2 Spike in Convalescent Individuals
Source: mBio. 2020 Oct 16;11(5):e02590-20. doi: 10.1128/mBio.02590-20 (PMC7569150; doi:10.1128/mBio.02590-20)
Supplement: TEXT S1 [file mBio.02590-20-s0001.docx]

**SUPPLEMENTAL MATERIAL AND METHODS**

**Ethics statement**

All work was conducted in accordance with the Declaration of Helsinki in terms of informed consent and approval by an appropriate institutional board. Convalescent plasmas were obtained from donors who consented to participate in this research project at Héma-Québec (REB # 2020-004) and CHUM (19.381). The donors met all donor eligibility criteria: previous confirmed COVID-19 infection and complete resolution of symptoms for at least 14 days.

**Plasmids**

The plasmids expressing the human coronavirus Spikes of SARS-CoV-2, SARS-CoV, NL63 229E (1, 2) and OC43 (3) were previously described. The D614G mutation was introduced using the QuikChange II XL site-directed mutagenesis protocol (Stratagene). The presence of the desired mutations was determined by automated DNA sequencing. The plasmid encoding for SARS-CoV-2 S RBD was synthesized commercially by Genscript. The RBD sequence (encoding for residues 319-541) fused to a C-terminal hexahistidine tag was cloned into the pcDNA3.1(+) expression vector. The vesicular stomatitis virus G (VSV-G)-encoding plasmid (pSVCMV-IN-VSV-G) was previously described (4).

**Cell lines**

293T human embryonic kidney cells (obtained from ATCC) were maintained at 37°C under 5% CO_2_ in Dulbecco's modified Eagle's medium (DMEM) (Wisent) containing 5% fetal bovine serum (VWR), 100 UI/ml of penicillin and 100µg/ml of streptomycin (Wisent). 293T-ACE2 cell line was previously reported (3).

**Protein expression and purification**

FreeStyle 293F cells (Invitrogen) were grown in FreeStyle 293F medium (Invitrogen) to a density of 1 x 10^6^ cells/mL at 37°C with 8 % CO2 with regular agitation (150 rpm). Cells were transfected with a plasmid coding for SARS-CoV-2 S RBD using ExpiFectamine 293 transfection reagent, as directed by the manufacturer (Invitrogen). One week later, cells were pelleted and discarded. Supernatants were filtered using a 0.22 µm filter (Thermo Fisher Scientific). The recombinant RBD proteins were purified by nickel affinity columns, as directed by the manufacturer (Invitrogen). The RBD preparations were dialyzed against phosphate-buffered saline (PBS) and stored in aliquots at -80°C until further use. To assess purity, recombinant proteins were loaded on SDS-PAGE gels and stained with Coomassie Blue.

**Plasma and antibodies**

Plasma from SARS-CoV-2-infected and uninfected donors were collected, heat-inactivated for 1 hour at 56 °C and stored at -80°C until ready to use in subsequent experiments. Plasma from uninfected donors were used as negative controls and used to calculate the seropositivity threshold in our ELISA and flow cytometry assays. The monoclonal antibody CR3022 was used as a positive control in ELISA assays and was previously described (5-7). Horseradish peroxidase (HRP)-conjugated antibody specific for the Fc region of human IgG (Invitrogen), the Fc region of human IgM (Jackson ImmunoResearch Laboratories, inc.) or the Fc region of human IgA (Jackson ImmunoResearch Laboratories, inc) were used as secondary antibodies to detect antibody binding in ELISA experiments. Alexa Fluor-647-conjugated goat anti-human IgG (H+L) Abs (Invitrogen) were used as secondary antibodies to detect sera binding in flow cytometry experiments.

**ELISA**

The SARS-CoV-2 RBD assay used was recently described (3). Briefly, recombinant SARS-CoV-2 S RBD proteins (2.5 μg/ml), or bovine serum albumin (BSA) (2.5 μg/ml) as a negative control, were prepared in PBS and were adsorbed to plates (MaxiSorp Nunc) overnight at 4°C. Coated wells were subsequently blocked with blocking buffer (Tris-buffered saline [TBS] containing 0.1% Tween20 and 2% BSA) for 1h at room temperature. Wells were then washed four times with washing buffer (Tris-buffered saline [TBS] containing 0.1% Tween20). CR3022 mAb (50ng/ml) or serial dilutions of plasma from SARS-CoV-2-infected or uninfected donors (1/100; 1/250; 1/500; 1/1000; 1/2000; 1/4000) were prepared in a diluted solution of blocking buffer (0.1 % BSA) and incubated with the RBD-coated wells for 90 minutes at room temperature. Plates were washed four times with washing buffer followed by incubation with secondary Abs (diluted in a diluted solution of blocking buffer (0.4% BSA)) for 1h at room temperature, followed by four washes. HRP enzyme activity was determined after the addition of a 1:1 mix of Western Lightning oxidizing and luminol reagents (Perkin Elmer Life Sciences). Light emission was measured with a LB941 TriStar luminometer (Berthold Technologies). Signal obtained with BSA was subtracted for each plasma and was then normalized to the signal obtained with CR3022 mAb present in each plate. The seropositivity threshold was established using the following formula: mean of all COVID-19 negative plasma + (3 standard deviation of the mean of all COVID-19 negative plasma).

**Flow cytometry analysis of cell-surface staining**

Using the standard calcium phosphate method, 10μg of Spike expressor and 2μg of a green fluorescent protein (GFP) expressor (pIRES-GFP) were transfected into 2 × 10^6^ 293T cells. At 48h post transfection, 293T cells were stained with plasma from SARS-CoV-2-infected or uninfected individuals (1:250 dilution). The percentage of transfected cells (GFP+ cells) was determined by gating the living cell population based on the basis of viability dye staining (Aqua Vivid, Invitrogen). Samples were acquired on a LSRII cytometer (BD Biosciences, Mississauga, ON, Canada) and data analysis was performed using FlowJo vX.0.7 (Tree Star, Ashland, OR, USA). The seropositivity threshold was established using the following formula: mean of all COVID-19 negative plasma + (3 standard deviation of the mean of all COVID-19 negative plasma + inter-assay coefficient of variability).

**Virus neutralization assay**

293T-ACE2 target cells were infected with single-round luciferase-expressing lentiviral particles. Briefly, 293T cells were transfected by the calcium phosphate method with the lentiviral vector pNL4.3 R-E- Luc (NIH AIDS Reagent Program) and a plasmid encoding for SARS-CoV-2 Spike (WT or D614G), SARS-CoV Spike or VSV-G at a ratio of 5:4. Two days post-transfection, cell supernatants were harvested and stored at –80°C until use. 293T-ACE2 target cells were seeded at a density of 1×10^4^ cells/well in 96-well luminometer-compatible tissue culture plates (Perkin Elmer) 24h before infection. Recombinant viruses in a final volume of 100μl were incubated with the indicated plasma dilutions (1/50; 1/250; 1/1250; 1/6250; 1/31250) for 1h at 37°C and were then added to the target cells followed by incubation for 48h at 37°C; cells were lysed by the addition of 30μl of passive lysis buffer (Promega) followed by one freeze-thaw cycle. An LB941 TriStar luminometer (Berthold Technologies) was used to measure the luciferase activity of each well after the addition of 100μl of luciferin buffer (15mM MgSO_4_, 15mM KPO_4_ [pH 7.8], 1mM ATP, and 1mM dithiothreitol) and 50μl of 1mM d-luciferin potassium salt (Prolume). The neutralization half-maximal inhibitory dilution (ID_50_) represents the plasma dilution to inhibit 50% of the infection of 293T-ACE2 cells by recombinant viruses bearing the indicated surface glycoproteins.

**Statistical analyses**

Statistics were analyzed using GraphPad Prism version 8.0.2 (GraphPad, San Diego, CA, (USA). Every data set was tested for statistical normality and this information was used to apply the appropriate (parametric or nonparametric) statistical test. P values <0.05 were considered significant; significance values are indicated as * P<0.05, ** P<0.01, *** P<0.001, **** P<0.0001.

**References**

1. **Hoffmann M, Kleine-Weber H, Schroeder S, Kruger N, Herrler T, Erichsen S, Schiergens TS, Herrler G, Wu NH, Nitsche A, Muller MA, Drosten C, Pohlmann S.** 2020. SARS-CoV-2 Cell Entry Depends on ACE2 and TMPRSS2 and Is Blocked by a Clinically Proven Protease Inhibitor. Cell **181:**271-280 e278.

2. **Hofmann H, Pyrc K, van der Hoek L, Geier M, Berkhout B, Pohlmann S.** 2005. Human coronavirus NL63 employs the severe acute respiratory syndrome coronavirus receptor for cellular entry. Proc Natl Acad Sci U S A **102:**7988-7993.

3. **Prévost J, Gasser R, Beaudoin-Bussières G, Richard J, Duerr R, Laumaea A, Anand SP, Goyette G, Ding S, Medjahed H, Lewin A, Perreault J, Tremblay T, Gendron-Lepage G, Gauthier N, Carrier M, Marcoux D, Piché A, Lavoie M, Benoit A, Loungnarath V, Brochu G, Desforges M, Talbot PJ, Gould Maule GT, Côté M, Therrien C, Serhir B, Bazin R, Roger M, Finzi A.** 2020. Cross-sectional evaluation of humoral responses against SARS-CoV-2 Spike. bioRxiv doi:10.1101/2020.06.08.140244**:**2020.2006.2008.140244.

4. **Lodge R, Lalonde JP, Lemay G, Cohen EA.** 1997. The membrane-proximal intracytoplasmic tyrosine residue of HIV-1 envelope glycoprotein is critical for basolateral targeting of viral budding in MDCK cells. EMBO J **16:**695-705.

5. **ter Meulen J, van den Brink EN, Poon LL, Marissen WE, Leung CS, Cox F, Cheung CY, Bakker AQ, Bogaards JA, van Deventer E, Preiser W, Doerr HW, Chow VT, de Kruif J, Peiris JS, Goudsmit J.** 2006. Human monoclonal antibody combination against SARS coronavirus: synergy and coverage of escape mutants. PLoS Med **3:**e237.

6. **Yuan M, Wu NC, Zhu X, Lee CD, So RTY, Lv H, Mok CKP, Wilson IA.** 2020. A highly conserved cryptic epitope in the receptor binding domains of SARS-CoV-2 and SARS-CoV. Science **368:**630-633.

7. **Tian X, Li C, Huang A, Xia S, Lu S, Shi Z, Lu L, Jiang S, Yang Z, Wu Y, Ying T.** 2020. Potent binding of 2019 novel coronavirus spike protein by a SARS coronavirus-specific human monoclonal antibody. Emerg Microbes Infect **9:**382-385.
